# Service delivery inaccessibility as a predictor of teenage pregnancy in South Africa

**DOI:** 10.4314/ahs.v23i3.9

**Published:** 2023-09

**Authors:** Mkwanazi Sibusiso

**Affiliations:** University of South Africa, Institute of Gender Studies

**Keywords:** Teenage pregnancy, service inaccessibility, multilevel modelling, South Africa, structural inequality, teenagers

## Abstract

**Background:**

With the onset of the South African democracy in 1994 it was hoped that many social inequalities would be addressed urgently. However, studies have shown that service delivery inaccessibility remains a challenge and investigating the social implications of such injustices remains important.

**Objective:**

This study determined to establish the association between service delivery inaccessibility and adolescent pregnancy in South Africa.

**Methods:**

Using data from 2019 and 2021 general household surveys, 7 737 teenage females were included. The study applied descriptive statistics, chi-squared testing as well as multilevel binary logistic regression.

**Results:**

Random-intercept multilevel binary logistic regression revealed that the risk of adolescent pregnancy independently increased as the level of service inaccessibility increased at household level (no services: OR=1.73, 1 service: OR=1.40, 2 services: OR=1.28) and community level (medium: OR=1.22, high: OR=1.38) at a P-value of 0.05.

**Conclusion:**

Findings highlight the need to guarantee universal service delivery urgently not only for development, but also to prevent adolescent pregnancy. Furthermore, the findings present evidence of structural factors driving adolescent pregnancy in South Africa, which renders continued cycles of poverty, injustice and early pregnancy amongst the majority of Blacks.

## Introduction

The indigenous people of South Africa have endured a long history of social injustice. This has resulted in their continual lack of adequate housing, amenities and decent employment. Since democracy in 1994 it was hoped that many of these atrocities would be addressed urgently. Instead in 2021, 9% of South Africans lived in informal settlements with houses made of non-permanent materials, according to the 2021 General Household Survey^1^.

Additionally, service delivery inaccessibility remains a challenge with approximately 66% of municipalities facing a crisis in 2010^2^. In the poorest squatter camps (as informal settlements are commonly termed in South Africa), individuals live like animals in temporary structure homes that are prone to fire and flooding, while sewerage and refuse waste run freely in the streets. The 2021 General Household Survey showed that 10% have no access to piped water,11% have no electricity and 35% have no sewerage system toilet facilities^1^.

Largely related, the incidence of service delivery protests in such disadvantaged localities seems to have increased in South Africa following democracy^3^. Nleya (2011) found that the occurrence of protests decreased as service delivery improved and service delivery directly and indirectly led to protest action through the perception of service delivery and living standards as well through the level of meeting attendance in a community^4^. Additionally, the study showed that half of the population in informal settlements were involved in protests as opposed to only 36% of people in formal settlements. This is home for many young South African females growing up in these poverty-stricken environments where crime and other dangers are present daily. To begin understanding the possible effects that such environments of injustice pose to teenage females this study investigates the association between teenage pregnancy and service delivery inequality in South Africa.

Teenage pregnancy remains an important health issue the world over with 11% of births resulting from 15–19-year-old females globally 5. The phenomenon occurs within developing nations, in 95% of cases, and is reportedly highest in sub-Saharan Africa at 109 births per 1000 females aged 15-19 years^6^,^7^. Although South Africa sports a lower prevalence of teenage pregnancy, compared to many African countries it has one of the highest levels within the Southern African region. Reddy in 2010 showed that24% of sexually active teenage females were ever pregnant in the South African youth risk behaviour survey^8^.

Dangerous obstetric and health consequences are associated with teenage pregnancy. Early childbearing increases the risk of obstetric fistulae, eclampsia, post-partum haemorrhage, sepsis, urinary tract infections, anaemia, malaria, puerperal endometriosis, episiotomy, depression and the risk of maternal mortality by five-fold^5^,^9^-^11^. Consequently, national government and non-government organisations have attempted to address this phenomenon through campaigns and legislation that increase access to contraceptives and termination of pregnancy, while criminalising child marriage and sexual relations between adults and minors^9^, ^12^-^14^. These initiatives have largely centred on the teenage female resulting in the policing of young women's bodies, politicians shaming pregnant teens on public platforms and, more recently, specific initiatives “to help wean young girls from sugar daddies” and “scholarships for virgins” 15.

Nevertheless, the adolescent female is not the only participant in this issue and the recommendations emanating from individual-level investigation, repeatedly, have not led to substantial lowering of pregnancy among this vulnerable group thus baffling researchers advocating for adolescent sexual and reproductive health. Furthermore, adolescent pregnancy level inconsistencies exist based on location sub-nationally, suggesting reasons beyond the individual level. Few studies have interrogated the possible risk posed by household or community level service delivery inequality where young women live.

One such study, conducted in Limpopo, was commissioned by the South African government “to identify and understand the psychosocial, economic, cultural and household factors associated with adolescent pregnancies” 16. The study showed that access to water and electricity were not significant predictors of adolescent pregnancy. These non-significant findings may be due to the location of the study and the resultant average socio-economic status, race pattern, and levels of service provision. If similar factors occurred among most adolescent females regardless of pregnancy status their effects would have been insignificant, as the study found. This may indeed be the case as Limpopo is known to be one of the poorer provinces in South Africa, with a rather homogenous racial and socio-economic profile 16.

The broken window theory, as posited by Wilson and Kelling (1982), guides this research 17. We hypothesise that increased service inaccessibility, at household and community levels, will be associated with a higher likelihood of teenage pregnancy. This hypothesis rests on the premise that individuals living in disorderly conditions will themselves behave disorderly, according to the above theory 17. Members of a household, which are recipients of good quality municipal services, consider themselves advantaged and could possess characteristics that reflect this advantage, as opposed to households that lack services and feel wrongfully neglected. In the context of this study, disorder can be associated with lack of municipal services namely, water, electricity, refuse collection and sewerage facilities. This may bring about a higher predisposition of risky and delinquent behaviour, such as unprotected sex leading to teenage pregnancy in such conditions. Wei et al's study conducted in 2005 revealed that physical disorder in the neighbourhood, including lack of refuse collection, increased the likelihood of teenage births in Pittsburgh 18.

## Materials and Methods

### Sources of data and procedure

The study utilised the revised general household surveys (GHS) of 2011, 2012 and 2013 as its main data source. These are secondary datasets where all details of participants were anonymised and replaced with a unique identity number to ensure confidentiality. These datasets are publicly available from the Statistics South Africa (Stats SA) database located at http://nesstar.statssa.gov.za:8282/webview/. Stats SA obtained ethical approval to collect the primary data and all survey participants gave voluntary consent to participate before commencement of data collection. The secondary data used for this data was de-identified and outcomes of this analysis do not allow re-identifying participants. The author asserts that all procedures contributing to this work comply with the ethical standards of the relevant national and institutional committees on human experimentation and with the Helsinki Declaration of 1975, as revised in 2008^19^.

The survey sample included individuals ranging from zero to 113 years. However, this paper's study population encompassed 73737 females aged 10 to 19 years: 4948 from 2019, and 2789 from 2021.These participants comprised 7454 young females who were not pregnant constituting 97% of the total sample, while 258 girls were pregnant. This represented 7810543 female teenagers who were not pregnant and 248743 pregnant teenage girls in the years 2019 and 2021 in South Africa. The study used secondary data where all details of participants were anonymised and replaced with a unique identity number to ensure confidentiality. Statistics South Africa obtained ethical approval to collect the primary data and voluntary consent from survey participants before commencement of data collection.

### Variable Identification

#### Dependent variable-Teenage Pregnancy in the past 12 Months

The study used a single measure for teenage pregnancy. In this paper teenage pregnancy is defined as pregnancy occurring below the age of 20 years. Individuals were allowed to self-report, if female, and were also allowed to report on behalf of other females within the same household “any female household member who had been pregnant during the past 12 months”. This was to make the question less threatening and more general in order to capture pregnancy in sensitive cases, for example for young girls within the household (1). Individuals' options to this item encompassed Yes, No, Do not know, Not applicable (for males) and Unspecified. Female household members who answered yes or whom other household members, from the above question, identifies and whose age was below 20, were classified as pregnant teenagers. Conversely, those that answered no and were not identified to have been pregnant by others yet, whose age was below 20 and above 10 were teenage females who were not pregnant. Teenage pregnancy was coded as 1 and teenage non-pregnancy as 0.

Independent variables encompassed the interest variables (service delivery inaccessibility at household and community levels) and controlling variables.

Household service delivery access index: level of access to municipal services for a household. These were coded as 0 for no services,1 for one service, 2 for two services and 3 for three or four services

Community level of service delivery inaccessibility: Community percentage of households with no municipal services divided into three equal categories of low, medium and high percentages.

The background variables were race, education level, employment status, orphanhood status, relationship to head of household, place of residence and province

**Race:** population groups Black, White, Coloured, Indian/Asian.

**Education level:** highest education level of respondent with the coding- No Schooling (0), Primary (1), Secondary (2), Tertiary (3)

**Employment status:** employment status of respondent coded as 1 if employed and 0 if unemployed

**Orphanhood status:** defined by mortality status of parents to encompass non-orphan

if parents are alive (0), paternal orphans (1), maternal orphans (2) or double orphans (3)

**Relationship to head of household:** respondent's relationship to the head of the household that can be Head (1), Immediate relative (2), Distant relative (3) or not related (4)

**Place of residence:** defined as either urban (2) or rural (1)

**Province:** geographical province where the respondent lives including Gauteng, Eastern Cape, North West, Northern Cape, Western Cape, Kwa-Zulu Natal, Mpumalanga, Free State and Limpopo

### Analysis

The study first described the entire study sample across the years, utilising frequency and percentage distributions. Annual percentages were calculated to show the level of teenage pregnancy, using 2011 to 2013 general household survey female adolescents. We calculated the adolescent pregnancy percentage through the following equation:


$$Teenage\;Pregnancy\;Percentage=\frac{Number\;of\;Pregnent\;Teenagers\;x}{Total\;Number\;of\;Female\;Teenagers\;(10-19\;years)}\times 100\ldots .(1)$$

Changes across time were tested for significance using the chi-squared test, as well as the chi-squared test for trend. The background characteristics of all pregnant teenagers were then described, as well as the levels of the interest variable phenomena amongst them. These bivariate relations were shown via tabulation and statistically verified through the chi-squared test. The level of significance for the chi-squared tests was 0.05. The above descriptive statistics were generated using STATA version 13.1.

The paper considered teenage pregnancy, a dichotomous outcome with possible responses of “yes” or “no”, through the use of regression. Random-intercept multilevel logistic regression tested the independent association between service delivery levels and teenage pregnancy in the past year. Four models were run in the STATA statistical programme to test the heterogeneity of adolescent pregnancy in different communities as well as to establish the association between service delivery inaccessibility and teenage pregnancy controlling for socio-demographic variables.

Multilevel modelling is a suitable statistical technique when individuals from the same households or geographical areas have the potential of being included in a study sample^20^. This, indeed, is the case for the general household survey as households from the primary sampling units (provinces) were sampled using systematic sampling, but every member within the household was interviewed. Therefore, the two-level model established the variation between individuals and individuals within the same communities in the risk of teenage pregnancy. Simple logistic regression would fail to capture this accurately as members within communities are similar, thereby violating the logistic regression assumption of independence of residuals^21^-^23^. This would result in underestimation of standard errors and very small p-values, making estimates of association appear falsely significant. Representation of the model follows:



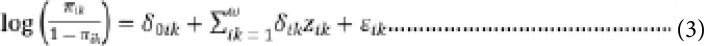



Where: π_ik_=probability of having been recently pregnant for the ith individual in the kth community – the dependent variable

δ_ik_ are the parameter coefficients of the model

z_ik_ are the independent regressors ε_ik_ are the residual errors

Results from multilevel analysis incorporated a fixed component and a random component. The fixed component of results accurately depicted factors associated with teenage pregnancy. These are presented as coefficient estimates with their associated standard errors, as well as the odds ratios for ease of interpretation through exponentiation of parameter coefficient estimates. A ratio greater than one implies that an individual in a given category would be more likely to experience teenage pregnancy, as opposed to an individual in the base category and vice versa^24^.

The random component of results quantified levels of heterogeneity between communities and indicated the extent to which unexplained community effects were present. We first ran an empty or null model that tested the significance and level of heterogeneity in adolescent pregnancy among communities in South Africa. This empty model also allowed us to determine the contextual influence on adolescent pregnancy^25^. We established intracluster correlation with the aid of the latent variable approach. This assumes that the underlying binary variable is a continuous latent variable yij with the variance at individual level being constant 26. The individual variance is assumed to have a standard logistic distribution, with mean 0 and variance of π2/3 =3.29. Therefore, according to this method the intracluster correlation due to level 2 is calculated using the formula:



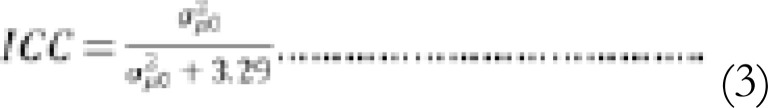



The empty model is specified as:



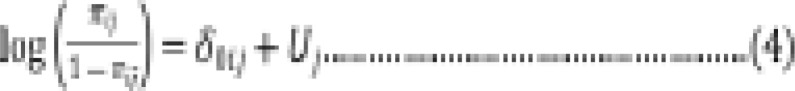



Where δ_0ij_ is the overall mean probability (prevalence) expressed on the logistic scale while U_j_ is the community level residual (having a normal distribution with mean 0 and constant variance $\sigma_{µ0}^{2}$. The significance of the between community variance was determined through the Wald test by diving the random intercept by its standard error. A random intercept variance is regarded as significant if the above division falls above 2.8 and below -2.8^20^.

## Results

### Descriptive Outcome

Teenage pregnancy was present over both years with 16369 cases, and 87373 cases respectively occurring nationally in 2019 and 2021. Over both years, a small proportion of study participants had been pregnant at 3.35%, while the majority of the teenage females were not pregnant. Chi-squared test results revealed that teenage pregnancy was statistically decreasing significantly over time with a p-value of 0.000. The chi-squared test for linear trend was significant with a p-value of 0.000. Therefore, there was a linear trend of teenage pregnancy from 2019 to 2021. This confirmed that teenage pregnancy had decreased linearly over time.

Females in the study sample had a median age of 15 years with an inter-quartile range of four years. Approximately 41% (40.92%) of the young females were aged 12 to 14 years, while 59.08% of them were aged 15 to 19 years. The majority of the study population was Black people at 89.22%, followed by Coloureds (7.34%), Whites (2.37%) and Indian/Asians that made up 1.07%. With regards to educational level, most of the study participants (52.54%) were attending secondary school, followed by a third (36.46%) attending primary school while 11% were not attending school.

**Figure 1:**
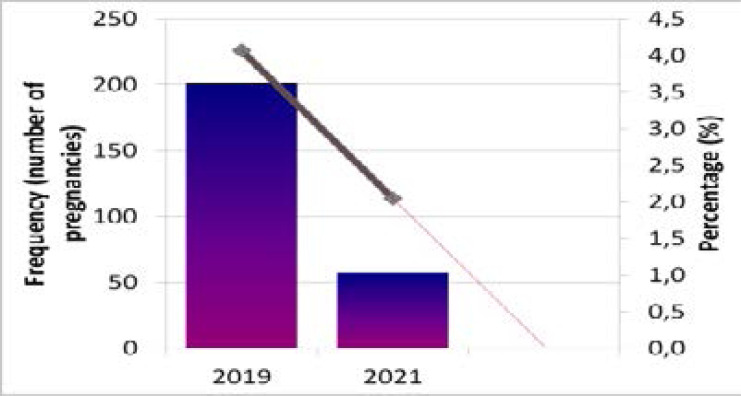
Teenage pregnancy incidence in South Africa- 2019- 2021 General Household Surveys Source: Authors' computation from 2019 & 2021 GHS

The greater part of study participants were unemployed, with only 1% working. Most of the study population had parents alive at 70.76% while 15.73% of participants were paternal orphans. Approximately 5.33% were maternal orphans and 5.66% were double orphans. Immediate relatives of the head of the household made up the largest proportion of study participants at 59.55%, 38.96% were distant relatives, 0.75% were heads of households and 0.75% of them were not related to the head of the household. The study population mostly comprised of individuals from urban areas (53.77%), while a lower proportion of individuals were from rural areas at 46.23%. The greatest proportion of study participants were from Kwa-Zulu Natal (21.03%), Gauteng (17.73%), Eastern Cape (15.20%) and Limpopo (13.40%). The levels of adolescent pregnancies for the 2019 and 2021 general household surveys are shown in figure 1.

The frequency of pregnancies per year decreased across time with 201 teenage girls experiencing pregnancy in 2019 while 57 girls in the sample were pregnant in 2021. Additionally, approximately, three percent (3.35%) of teenage females had given birth in the previous twelve months over the two years. We added a linear forecast line to establish the current trend of pregnancy and predict this into the future. As seen in figure 1, it is expected that pregnancy amongst teenage females in South Africa will decrease, based on the 2021 data.

The distribution of pregnant teenage females' background characteristics is shown in table 1 below. Levels of pregnancy in the various categories of characteristics were marginally or significantly different exhibiting a p-value of less than 0.1 using the chi-squared test. The distribution of pregnancy differed significantly by age group, with only 0.22% of younger adolescents aged between 10 to 14 years being pregnant across the years while almost 6% of 15–19-year-olds were pregnant in the preceding year. Regarding race, Whites and Indians experienced the lowest proportions of teenage pregnancy compared to other races. However, Blacks had the highest levels of pregnancy at 3.43%.

**Table 1:** Bivariate analysis of teenage pregnancy by study characteristics, 2019 and 2021 General Household Surveys

Characteristics	Pregnant n=258	P-value
**Age Group**		0.00
10–14-year-olds	0.22	
15–19-year-olds	5.50	

**Race**		0.19
African/Black	3.43	
Coloured	3.35	
Indian/Asian	0.00	
White	1.64	

**Educational Level**		0.00
Primary	0.25	
Secondary	2.62	
Not Attending School	17.08	

**Employment Status**		0.00
Unemployed	3.26	
Employed	16.67	

**Orphanhood Status**		0.00
Double Orphan	4.38	
Paternal Orphan	4.95	
Maternal Orphan	3.88	
Parents Alive	2.84	

**Relationship to Head of Household**		0.23
Head	6.90	
Immediate Relative	3.50	
Distant Relative	3.00	
Not Related	5.17	

**Place of Residence**		0.06
Rural	3.76	
Urban	2.99	1

The percentage of pregnancy was highest among teenage females who were not attending school at 17.08%, followed by those with secondary education (2.62%) and primary education (0.25%). Teenage females who were employed had significantly higher proportions of pregnancy at 16.67% than those who were unemployed. Teenage pregnancy was generally higher among orphans regardless of the type compared to teenage females with both parents alive. Maternal orphans (3.88%), double orphans (4.38%) and paternal orphans (4.95%) had the largest proportions of pregnant teenage females. Regarding the relationship to the household head, pregnancy occurred most among girls who were household heads at 6.90%, followed by teenage females who were not related to the household head (5.17%). In contrast, the lowest proportion of pregnancy occurred among teenage females who were distant relatives to the head of the household at 3.00%. Finally, considering place of residence, the occurrence of pregnancy was higher among teenage females residing in rural settings at 4.56%.

**Figure 2:**
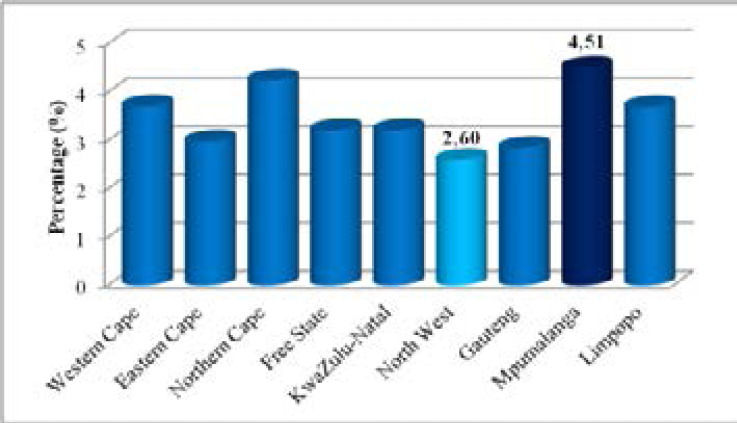
Teenage pregnancy in provinces of South Africa, 2019 and 2021 GHS Source: Authors' computation from 2019 & 2021 GHS

**Figure 3:**
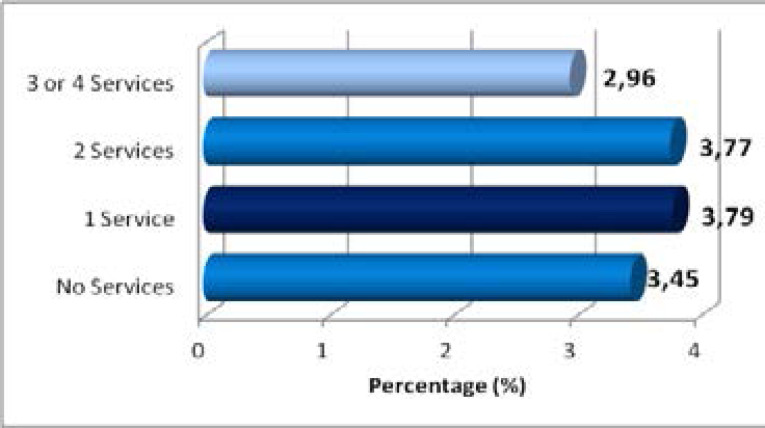
Teenage pregnancy by household services in South Africa:2019 and 2021 GHS Source: Authors' computation from 2019 and 2021 GHS

**Figure 4:**
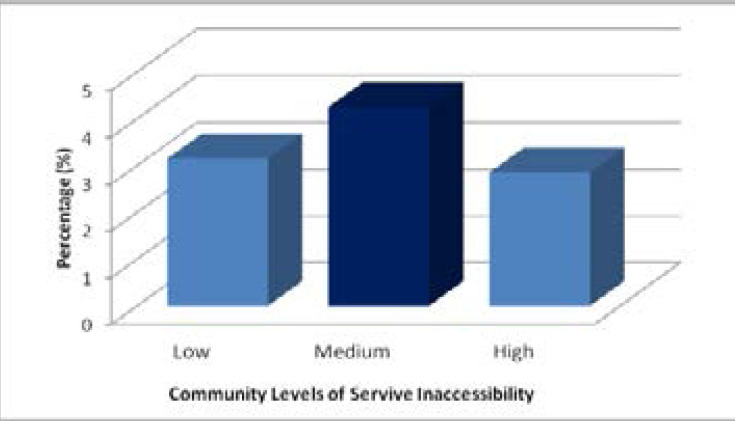
Teenage pregnancy by community levels of service delivery inaccessibility in South Africa:2019 and 2021GHS Source: Authors' computation from 2019 & 2021 GHS

As seen in figure 2, the levels of teenage pregnancy differed across provinces in South Africa. In particular, the highest percentage of teenage pregnancy was amongst females residing in Mpumalanga at 4.51% whereas the lowest levels were among teenage females from the North West at 2.60%. The chi-squared test showed that these numerous differences in pregnancy levels were statistically significant with p-values of less than 0.05 for age group, educational level, employment status and orphanhood status. We then considered the levels of teenage pregnancy across the interest variables as seen in figures 3 and 4.

The household service delivery index showed higher levels of pregnancy amongst teenage females living in homes with fewer services and lower levels of pregnancy among those living in homes with more services. The lowest levels of pregnancy were among females living in homes with access to three or more services with 2.96% being pregnant. Figure 4 shows the percentage of teenage pregnancy for females living in communities with different levels of service delivery inaccessibility. The figure shows that highest levels of pregnancy occurred amongst teenage females living in communities with medium service inaccessibility.

### Inferential Outcome

Fixed Effects: The results of the unadjusted and hierarchically adjusted multilevel logistic regression modelling are shown below in table 2. Firstly, for household service delivery inaccessibility at household level, findings revealed a consistent positive relationship, with the likelihood of teenage pregnancy increasing as inaccessibility increased. In unadjusted analysis the effect is substantial: 15% higher odds among teenage females living in homes with two services, 30% higher average odds for those living in homes with one service and 93% higher average odds for teenage females residing in households with complete inaccessibility to services as compared to their counterparts from homes with access to three or four services.

**Table 2:** Unadjusted and adjusted incremental multilevel logistic regression (GHS 2019 &2021

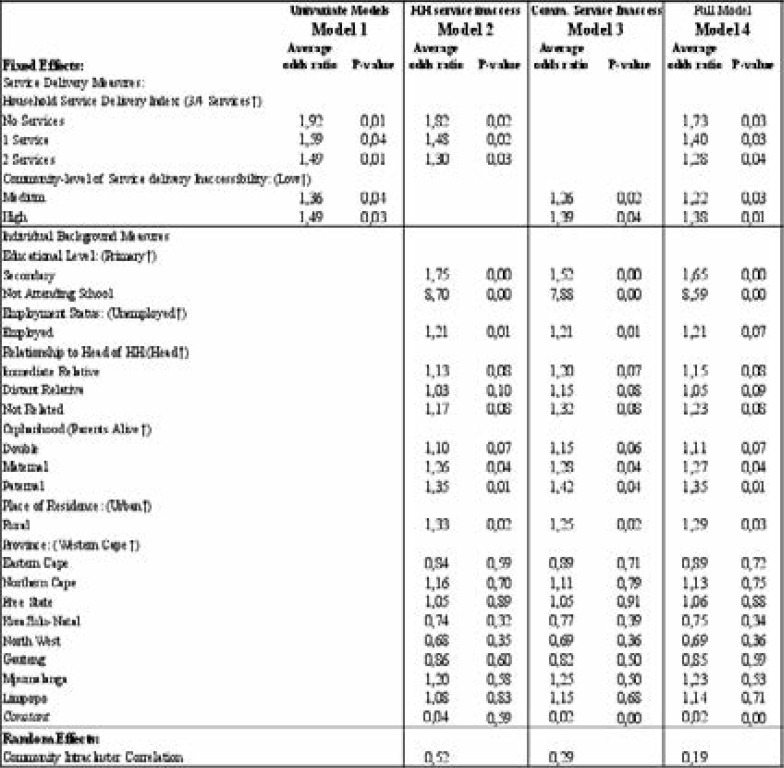

This general result of increasing likelihood of the outcome with household service delivery inaccessibility remained fairly consistent, even upon adjusting for control background factors as seen in Model 2, as well as when community-levels of service delivery inaccessibility was controlled for, as seen in Model 4.

Similarly, at community-level, higher levels of service inaccessibility were associated with higher average odds of teenage pregnancy. Specifically, at univariate level, teenage females living in communities with medium levels of service inaccessibility had a 36% higher average chance of experiencing pregnancy, while those from communities with high inaccessibility had 49% higher average odds of pregnancy. Upon controlling for individual-level characteristics, the association remained statistically significant, yet decreased slightly to 26% and 39% higher average likelihood, as seen in Model 3, respectively. In the final model that allowed controlling for teenage characteristics as well as household-level service inaccessibility, females living in communities with medium levels of service delivery inaccessibility had 22% higher average odds of teenage pregnancy, while those from communities with high service delivery inaccessibility had 38% higher average likelihood of early pregnancy. Once again, these effects were significant at univariate level and having adjusted for background and household-level characteristics.

### Random Effects

The empty model (not shown in table 2) allowed us to establish the levels of variability in teenage pregnancy across communities. The odds of pregnancy for a teenage female from an “average” community (µ0j = 0) was 0.09. Therefore, a teenage female picked at random from an average community was less likely to be pregnant. The community variance was statistically significant at the 5% level of significance and showed that the log odds of teenage pregnancy differed between communities by 54%. The intracluster correlation was statistically significant and at 0.134, signifying that teenage females from within the same communities were 13% similar, with regards to the likelihood of pregnancy. This increased as more variables were controlled for. In the final model that controlled for all factors, females within the same community were 19% similar, with regards to the likelihood of pregnancy.

## Discussion

The study determines the association between household and community levels of service delivery inaccessibility and teenage pregnancy in South Africa. Studies acknowledge that access to basic amenities is an important predictor of adult, child and maternal health^27^, ^28^. Nevertheless, there are limited studies that investigate the influence of service delivery inaccessibility on teenage pregnancy and even fewer that can confirm the findings presented in this study. Our results show that service delivery inaccessibility was significantly associated to teenage pregnancy, with the likelihood of teenage pregnancy increasing as inaccessibility rose within households and communities.

Decker et al (2018) and Wei et al. (2005) found that the physical disorder index was associated significantly with rates of teenage births^18^, ^29^. Teenage pregnancy increased as the index in physical disorder rose in this study conducted in Pittsburgh, Pennsylvania. A Limpopo study found access to amenities decreased the likelihood of unwanted teenage pregnancy yet the results were not statistically significant 16. In particular, the study investigated the access to water and electricity separately. Notably, the study failed to use multilevel modelling in regression analysis. Similarly, the World Health Organisation (2019) found that in resource-poor settings, fertility rates are higher^30^. Although Bradshaw et al (2005) found that lower conception rates occurred in areas with low access to services, they explained that this was due to lack of services occurring more in rural areas where teenage conception was low due to other rural factors in England^31^.

South Africa continues to suffer from the legacies of apartheid regarding service delivery inaccessibility. During the apartheid regime people were classified and allocated or denied access to resources, including basic amenities, according to the colour of their skin and hair texture. As a result, areas where White people lived had access to all services, while areas inhabited by Blacks, Coloureds and Asians had fewer services^32^. Upon the official downfall of apartheid in 1994, a new democratic government was instated yet the social inequalities remain to this day. In 2010, approximately 66% of municipalities across the country faced a service delivery crisis, while Masiya et al (2019) found that 44% of individuals were dissatisfied with service delivery nationally^2^, ^33^. This has been blamed on skills shortages, lack of accountability, corruption and dubious decision-making at local government level. The following section will discuss the numerous ways that service delivery inaccessibility may possibly be related to teenage pregnancy.

There are several reasons that could explain the association found between service delivery inaccessibility levels and teenage pregnancy in this study. Firstly, the lack of access to basic services has been used as a means of proxying socio-economic status in previous research 34. This is based on the foundation that as the level of access to amenities increases so would socio-economic status. Richter et al (2009) state that investigating the effect of social and economic conditions on adolescent health assists in supporting developmental and ecological agendas^35^.

In studying health inequalities, socio-economic measures need to be sensitive enough to capture logical hierarchy. Additionally, they should not be a result of health status as reverse causation may make interpretation of results difficult^36^. The lack of basic amenities has limitations in this regard as a socio-economic measure because it may influence levels of morale and self-reported health. However, teenage pregnancy would not be affected by this issue. Further, Bärnighausen et al. (2007) concluded that household asset indices were valid proxies of wealth in health surveys conducted in rural Africa^37^. To this end, certain studies have gone on to define urban poverty as the deficiency of three basic amenities viz. electricity, flush toilet and piped water^38^.

Socio-economic status has been defined as “the relative position of a family or individual on a hierarchical social structure, based on their access to or control over wealth, prestige and power” 39.

Socio-economic status has been said to affect health in three main ways. The first relates to materialistic privilege where individuals with higher earnings can have superior nutrition, living conditions and access to health 36. The second considers differences in behaviour and knowledge, where higher socio-economic status affects cognitive skills and knowledge, leading to the appropriate use of health care. Finally, higher socio-economic status increases empowerment, social status and integration to increase independence in important areas of life, such as relationships.

With regards to teenage pregnancy, individuals from a higher socio-economic status would have better access and knowledge of contraceptives, as well as a higher ability to negotiate and enforce safe sex with their partners. Studies have shown that teenage women from poor homes are more likely to have premarital births than those from affluent backgrounds^40^-^44^. Consequently, Bradshaw et al. (2005) and Adeyinka et al (2019) advised that spatial assessments of teenage conception should consider poverty^31^, ^45^. They found a statistically significant association between the rate of teenage conception rates and a few deprivation realms with deprivation accounting for more than 75% of variation in teenage conception rates.

Specific links of socio-economic status with teenage pregnancy also exist due to the characteristics of families and parents in poor communities. Firstly, Nettle and Cockerill (2010) as well as Nomaguche and Milkie (2020) proposed that individuals whose parents invest less emotional support in them wanted children at younger ages^46^,^47^ . Poor parents give less positive attention to their children due to their own time and money constraints that keep them stressed^48^.

Secondly, oblique intergenerational transmission occurred through individuals in communities with younger parents wanting children at a younger age as well. Brady (2019), Phillips (2018) and Wilson (1991), clarify that poor areas lack educated, employed, and married role models, which perpetuates social welfare reliance and family instability as norms^49^-^51^. This leads to poor families giving up hope of ever overcoming economic hardship for themselves or their children. Schooling and employability may be so weak in such areas that staying in school and avoiding early pregnancy may not be advantageous 52. Accordingly, families living in poverty may adopt practices that are less conducive to scholarly and career success while encouraging premarital childbearing^53^. Furthermore, individuals born to younger parents wanted children at a younger age- a process termed vertical intergenerational transmission^46^.

Premarital childbearing occurring in poor areas is related to a higher predisposition to mortality. Poverty has been shown to predict adverse health, injury and mortality 18. Dinh et al. (2022) and Wilson and Daly (1997) posit that accelerated reproduction motivation occurs through psychological mechanisms in neighbourhoods that experience high mortality^54^, ^55^. These breeds ideas of the environment being unsafe and lowers the expectation for long healthy life, making premarital reproduction in such areas a sub-conscious, but rational choice of survival^56^.

Therefore, from an evolutionary perspective, shorter life, social learning, contextual prompts, and high mortality regimes work together to increase costs and lower benefits in delaying motherhood. Such benefits entail “higher quality” children, who achieve more in life. However, if the realisation and economic returns of schooling and career advancement are limited, as is the case in poor areas, pre-reproductive accumulation of resources cannot be achieved. Hence the process of attaining children of “higher quality” is futile^56^. It follows that theory predicts individuals with a short reproductive life span to follow a “fast” life-history of early motherhood, low investment in children and high fertility rate 57.

Numerous studies looking at inaccessibility of amenities have focused on slums and urban poor areas^58^-^60^. This is mainly because increased urbanisation in the African setting has been associated with the mushrooming of informal settlements, also known as slums. Such communities are characterised by make-shift houses made of corrugated iron sheets, without access to electricity, sanitation services, refuse removal and piped water. Similarly, in this study, 95% of the households without access to all services and 85% of the households with access to only one service were from urban areas. A study conducted in Kenya concluded that residents of urban slums in Africa may suffer more from the effects of their residential spaces, due to the lower ability of governments in developing countries to support the health, education and social welfare of individuals 61. It further stated that greater financial support may mitigate the influence of slum residence on risky behaviour in such communities. Inequality is said to be highest in African urban areas and by 2025, it is estimated that 60% of young people from developing countries will reside in cities, yet mostly be poor^35^. Therefore, the welfare of urban adolescents is a growing concern for developing countries, South Africa included.

In light of the study's findings, we consider a number of recommendations to decrease teenage pregnancy in South Africa. This study has found that levels of service inaccessibility are linked to teenage pregnancy. From this, we can then plot areas that are more prone to teenage pregnancy and guarantee that they are targeted for intervention programmes against teenage pregnancy. These programmes need to focus on poverty alleviation, to ensure any risk due to low socio-economic status is eradicated. Additionally, the programmes should involve assessment of the distance, quality, and quantity of youth-friendly reproductive services in the area and provide such if they are far, not adequately resourced or lacking in quality. Providing these services at school may yield the most optimum results.

In assessing such areas, it is also pertinent to check the presence and quality of supervised recreational activities and centres. Coordinated book clubs, competitive community or church affiliated sport clubs and other recreational activities, which are monitored by adults or older youth, will be beneficial in increasing involvement in community activities and decreasing time available for relationships and sex among this group of growing minds. Obtaining sponsorship for such activities and partnering with NGOs already involved in youth development programmes will assist this process further.

It is pertinent that government prioritises the provision of affordable subsidised housing with essential services present to deal with the environments that young people grow up in. The above provision should be followed by demolishing of urban poor settlement areas with no amenities, to avoid their repopulation. Government should plan adequately to implement the same provision of quality housing in rural areas and develop rural areas to ensure economic activity in these areas as well.

Furthermore, non-governmental organisations could consider setting up programmes that conduct home visits, to check living conditions and identify homes needing urgent assistance regarding financial assistance, social worker intervention, counsellors, rehabilitation services, etc. This may ensure that teenagers grow up in safe, encouraging homes and communities. Teenage pregnancy preventative programmes should fortify prevention in a supportive manner for individuals living in amenity-deprived communities. Programmes could include building parks near informal settlements and townships, providing transport to school, and ensuring that nurses visit schools regularly and have sex talks for adolescents.

To correctly understand the pathways of service delivery inaccessibility dynamics and their link to teenage pregnancy in South Africa, it is imperative, additionally to conduct qualitative studies in answering the “how and why” research questions pertaining to this phenomenon. As authors, we continuously debated the relevance of previous literature while writing the pathways that underlie some of the associations found in this study. For example, the whole notion of socially disorganised and poorer communities having less levels of social cohesion and collective efficacy, may not be the case in our setting. Many poor South African communities exhibit high levels of social interaction, communitarianism and working in unity to act against common problems. Communities that are situated in rural areas and urban townships display these characteristics. This has resulted in the establishment of communal security groups, addressing government collectively and other community initiatives. Therefore, it is important to determine what are the other issues at hand in the South African setting that social disorganisation-related factors operate through that international studies have not and cannot highlight because of the different context that we are in.

In conclusion, although teenage pregnancy is traditionally painted as an individual-level issue, primarily placing the responsibility of prevention on the young female, it is in fact a public issue due to its creation by the physical and social environments that young females grow up in. This paper has shown that increasing local and national government provision of basic amenities is needed to facilitate the improvements in general health, as well as lower teenage pregnancy. However, these services would need to accompany supply of social protection, poverty relief and job-creating opportunities to improve the overall quality of life, comprehensively, in such communities 35. Since the welfare of urban adolescents is a growing concern for developing countries, South Africa included, it is imperative that the provision of basic amenities to all citizens of South Africa become an urgent governmental pursuit.
